# Supplementary material for: Strategies for robust renovation of residential buildings in Switzerland
Source: Nat Commun. 2024 Mar 12;15:2227. doi: 10.1038/s41467-024-46305-9 (PMC10933253; doi:10.1038/s41467-024-46305-9)
Supplement: Supplementary file 1 — Supplementary Information [file 41467_2024_46305_MOESM1_ESM.pdf]

Supplementary information

Strategies for robust renovation of residential buildings in Switzerland

Alina Galimshina[*1], Maliki Moustapha[2], Alexander Hollberg[3], Sébastien Lasvaux[4], Bruno Sudret[2], Guillaume Habert[1]

[1] ETH Zürich, Institute of Construction and Infrastructure Management (IBI), Chair of Sustainable Construction, Stefano-Franscini-Platz 5, 8093 Zürich, Switzerland
[2] ETH Zürich, Institute of Structural Engineering (IBK), Chair of Risk, Safety and Uncertainty Quantification, Stefano-Franscini-Platz 5, 8093 Zurich, Switzerland
[3] Chalmers University of Technology, Department of Architecture and Civil Engineering, Sven Hultins Gata 6, 412 96 Göteborg, Sweden
[4] University of Applied Sciences of Western Switzerland (HES-SO), School of Business and Management Vaud (HEIG-VD), Institute of Energies (IE), Avenue des Sports 20, Yverdon-les-Bains 1401, Switzerland

Table of contents:

1. Analyzed buildings' structure and energy performance 1
2. Description of uncertain parameters 2
3. Renovation solutions description 3
4. Robust optimization 5
5. Building stock generalization 6
6. The resulting renovation options for conventional and non-conventional materials 8
7. Initial energy consumption and LCA, LCCA and insulation thickness of the renovated solutions for gas and heat pump heating systems 10
8. Electricity mix scenarios 12

1. Analyzed buildings' structure and energy performance

Existing state of the analyzed buildings can be seen in Table S 1.

Table S 1 – existing state of analyzed buildings

Building	1	2	3	4	5	6
Year of construction	1911	1939	1960	1970	1972	1988
Construction period	Before 1919	1919-1945	1946-1960	1961-1970	1971-1980	1981-1990
Heated floor area, m^2	1563	2445	1475	2811	1446	5215
Exterior walls structure	Stone, not insulated, U = 1.60 W/(m²K)	Hollow brick, not insulated, U-value – 1.22 W/(m²K)	Hollow brick, not insulated, U-value – 0.65 W/(m²K)	Reinforced concrete, not renovated, U-value – 1.53 W/(m²K)	Double brick wall, 4 cm of mineral wool, U-value – 0.55 W/(m²K)	Reinforced concrete, 8 cm of mineral wool, U-value – 0.43 W/(m²K)
Roof structure	Wooden beams, 10 cm	Hollow brick, not insulated,	Reinforced concrete. 10 cm	Reinforced concrete, 10 cm EPS, U-	Reinforced concrete, 6 cm mineral	Reinforced concrete, 10 cm EPS, U-

	rockwool, U = 0.98 W/(m²K)	U-value – 1.02 W/(m²K)	rockwool, U-value – 0.31 W/(m²K)	value – 0.36 W/(m²K)	wool, U-value – 0.50 W/(m²K)	value – 0.47 W/(m²K)
Ground slab structure	Concrete, not insulated, U = 2.80 W/(m²K)	Hollow brick, not insulated, U-value – 0.98 W/(m²K)	Hollow brick, not insulated, U-value – 1.06 W/(m²K)	Reinforced concrete, not insulated, U-value – 2.14 W/(m²K)	Reinforced concrete, not insulated, U-value – 1.53 W/(m²K)	Reinforced concrete, not insulated, U-value – 2.18 W/(m²K)
Storeboxes	-	Hollow brick, not insulated, U-value – 2.66 W/(m²K)	Hollow brick, 10 cm, U-value – 3.50 W/(m²K)	Reinforced concrete, not insulated, U-value – 4.00 W/(m²K)	Brick, not insulated, U-value – 0.99 W/(m²K)	Concrete slab, 6 cm mineral wool, U-value – 0.49 W/(m²K)
Windows	Double glazing with PVC frame, U-value 1.60 W/m^2,K	Double glazing, PVC frame. U-value 1.94 W/m^2,K	Double glazing, wooden frame, U-value 2.95 W/m^2,K	Double glazing, PVC frame, U-value 2.33 W/m^2,K	Double glazing with low-E layer, PVC frame, U-value 1.70 W/m^2,K	Double glazing, PVC frame, U-value 2.70 W/m^2,K
Heating demand, deterministic, SIA 380/1, kWh/m^2,a	141	94	110	109	91	88

2. Description of uncertain parameters

The description of the uncertain parameters including range and distribution type can be seen in Table S 2.

Table S 2: Description of uncertain parameters

**Model parameter**		**Parameters**	**Moments**	**Distribution**	**Source**
Embodied LCEI $(m_{production})$ and investment costs $(C_{investment})$					
**Embodied impact gas boiler [kgCO_2eq.]**		[2246, 3370]		uniform	[1]
**Embodied impact wood pellets boiler [kgCO_2eq.]**		[915.9, 1375]		uniform	
**Embodied impact heat pump [kgCO_2eq.]**		[4600, 5520]		uniform	
**Cost gas boiler [CHF/ERA]**		[30.1, 45.2]		uniform	[2], [3]
**Cost wood pellets boiler [CHF/ERA]**		[37.7, 56.5]		uniform	
**Cost heat pump [CHF/ERA]**		[40.7, 61]		uniform	
**Embodied impact components [%]**		[-30,30]		uniform	[4], [5]
**Investment cost components [%]**		[-20, 20]		uniform	[3]
Operational environmental and cost inputs					
**Thermal energy generation - $k_{op.}$ [kgCO2-eq./kWh]**	Gas	[0.248, 0.249]		uniform	[4], [5]
	Wood pellets	[0.038, 0.048]		uniform	
	Heat pump	[0.036, 0.039]		uniform	
**Operational cost for heating [CHF/kWh] m_{op}**	Gas	[0.101, 0.111, 0.127]		triangular	[2], [6]
	Wood pellets	[0.095, 0.107, 0.13]		triangular	
	Heat pump	[0.064, 0.079, 0.093]		triangular	
**Inflation rate r [%]**		[0.5,2]		uniform	[7]
**Discount rate (real) $d_{nominal}$ [%]**		[2.5,4.5]		uniform	[3]

Components reference service life RSL [years]				
**Exterior wall [years]**		[40.6, 11.6]	lognormal	[8]
**Roof [years]**		[30.4, 9.6]	lognormal	
**Slab [years]**		[33.7, 14.2]	lognormal	
**Wall against unheated surface [years]**		[40.6, 11.6]	lognormal	
**Windows [years]**		[27.5, 12.2]	lognormal	
**Oil boiler [years]**		[19.4, 3.1]	lognormal	
**Gas boiler [years]**		[18.8, 3.3]	lognormal	
**Wood pellets boiler [years]**		[18.3, 2.8]	lognormal	
**Heat pump [years]**		[17.1, 6.4]	lognormal	
**Electric boiler [years]**		[19.8, 5]	lognormal	
**Slab against unheated surface [years]**		[33.7, 14.2]	lognormal	
**Roof against unheated surface [years]**		[30.4, 9.6]	lognormal	
System performance				
**Existing windows U-value U_{ex} [W/m²*K]**		[2.9, 0.58]	lognormal	Assumption, [9]
**Existing exterior wall degradation d_i [%]**		[10, 3]	gumbel	Assumption [10]
**Existing roof insulation degradation d_i [%]**		[20, 5]	lognormal	Assumption [10]
**Thermal bridge renovation φ [%]**		[18, 5]	gaussian	Assumption
**Efficiency loss of the existing system [%]**	[0.15, 0.25]		uniform	Assumption [11]
**Efficiency loss of a new system [%] PF**		[0.15, 0.05]	gaussian	Dependent on the heating system, shown in SI1
**Existing slab against unheated surf., degradation [%] d_i**		[10, 5]	lognormal	Assumption [12]
User-oriented parameters				
**Operating temperature inside T_{in} [°C]**	[20,23]		uniform	[13]
**Building occupation schedule t_{occ} [h/day]**	[8, 16]		uniform	+/- 4 hours to the suggested 12 h value by [14]
**Airflow q_{vent} [m³h/m²]**	[0.7, 1]		uniform	[13]

3. Renovation solutions description

This section represents the renovation scenarios that were considered in the model.

In regards to the renovation methodology and thermal performance selection, the current approach includes the range from uninsulated states to attaining the U-value target specified by SIA 380/1 (0.25 W/(m²·K)). Subsequently, we aimed to meet the punctual requirement qualifying for subsidies from the Gebaudeprogram (0.2 W/(m²·K)), alongside targeting an extreme scenario ≤ 0.17 W/(m²·K) in line with Minergie building certification criteria. We also examined cases falling within these defined parameters to precisely capture optimal renovation strategies. The non-renovated scenarios for all components are also included in the study and the insulation thickness is based on available market options. The precise

insulation thickness is varying for each case study due to the varying heat loss coefficient of the initial elements.

In the Table S 3 and Table S 4, characteristics of conventional and non-conventional insulation materials as well as substructures are presented. In the Table S 5, windows types and qualities are presented. Table S 6 represents the cladding types.

Table S 3: Conventional insulation materials applied in the study

Materials	Thickness (m)	Thermal conductivity (W/m,K)	Density (kg/m^3)
Glasswool + solid wood	0.04	0.034	50
	0.05		
	0.07		
	0.08		
	0.12		
	0.16		
	0.23		
Rockwool	0.05	0.035	60
	0.07		
	0.08		
	0.1		
	0.11		
	0.16		
	0.23		
Cellular glass	0.1	0.06	120
	0.15		
	0.25		
	0.38		
Wood fibre + solid wood	0.04	0.038	50
	0.09		
	0.22		
	0.38		
Hagatherm aerogel	0.02	0.02	150
	0.04		
Multipor	0.05	0.045	90
	0.08		
	0.12		
	0.15		
EPS	0.05	0.03	40
	0.1		
	0.15		
	0.2		
	0.3		
Cellulose fibre + solid wood	0.13	0.04	35
	0.16		
	0.21		

Table S 4: Non-conventional insulation materials applied in the study

Materials	Thickness (m)	Thermal conductivity (W/m,K)	Density (kg/m^3)
Wood fibre + solid wood	0.06	0.038	50
	0.1		
	0.12		
	0.2		
Hempcrete	0.08	0.07	600
	0.2		

	0.3		
	0.38		
Hemp mat + solid wood	0.03	0.04	37
	0.06		
	0.12		
	0.18		
	0.22		
Strawbale + solid wood	0.2	0.066	105
	0.48		
	0.7		

Table S 5: Windows' types and properties

Type	U-value	Frame	Glazing
1	$U_{frame} = 1.24$ $U_{glazing} = 1.1$	Wood	Double
2	$U_{frame} = 1.24$ $U_{glazing} = 0.6$	Wood	Triple
3	$U_{frame} = 0.94$ $U_{glazing} = 0.6$	PVC	Triple
4	$U_{frame} = 0.94$ $U_{glazing} = 0.5$	PVC	Triple

Table S 6: Cladding types applied in this study

Categories	Cladding type
External walls	Solid wood
Surfaces against unheated spaces (wall, floor)	Gypsum plaster
Ceiling (against attic)	Medium density fibreboard (MDF)

4. Robust optimization

This section outlines the methodology adopted to identify the optimal and robust solution for building renovation, accounting for uncertain parameters that are described above.

The optimization focuses on two key quantities: the total costs and the greenhouse gas emissions throughout the building's life cycle. Robust optimization is applied to account for the inherent variability in the input parameters and consequently in the associated quantities of interest (QoI). The objective is to identify the optimal solution that exhibits minimal sensitivity to input variations. A multitude of robustness metrics have been proposed in the literature, where the mean and standard deviation, either individually or in tandem, emerging as the most widely utilized measures [15], [16]. In this study, we examine conservative quantiles as an indicator of robustness. Such an indicator can be seen as a combination into a single metric of both the mean μ and standard deviation σ in the form $\mu + k\,\sigma$, where k is a positive factor controlling the degree of robustness of the solution. We estimate the quantiles using crude Monde Carlo simulation [17]. The idea of the multi-objective robust optimization problem revolves around the objective of diminishing the 90th percentile of combined costs and greenhouse gas emissions across a range of design parameter options. This is performed by using the non-dominated sorting genetic algorithm II (NSGA-II), a widely-used state-of-the-art multi-objective optimization algorithm [18]. This algorithm is especially suitable, as it can be effortlessly adjusted to manage the mixed nature of continuous-categorical variables that arise in the specific issue we are addressing.

Nonetheless, its primary limitation lies in its computational burden, given that it necessitates repetitive assessments of the objective functions, specifically the quantiles of the two QoI in this context. Additionally, the assessment of quantiles for various design parameter combinations involves the propagation of input uncertainties through the computational model of the integrated LCA and LCCA, achieved through a basic Monte Carlo simulation approach. The cumulative expense incurred by both uncertainty propagation and the evaluation of objective functions renders the overall cost of the optimization process prohibitive.

To reduce the computational power needed for the analysis, the utilization of surrogate modeling is considered, with a specific focus on Gaussian process modeling, commonly referred to as Kriging. Kriging stands out as a widely used technique that operates under the assumption that the function being approximated is a realization of a Gaussian process [19], [20]. Statistical learning is employed to calibrate the surrogate model using a constrained series of evaluations of the original model, which is commonly referred to as the experimental design. The size of the experimental design is usually relatively small, i.e. in the order of tens or a few hundreds of samples. After the calibration and construction of a surrogate model, it becomes possible to assess it numerous times within a relatively brief timeframe, typically on the order of seconds. Subsequently, this enables the execution of NSGA-II by substituting the original model with the constructed surrogate. The reliability of the consequent outcomes greatly depends on the precision of the surrogate model. In this study, the latter is dynamically constructed by regulating its localized precision during the optimization process, thereby enabling us to guarantee the quality of the identified Pareto front. Detailed description of the methodology can be found in Moustapha et al [21].

5. Building stock generalization

This section delves into the potential for extrapolating the findings of this study to the overall energy consumption related to the building stock in Switzerland. It explores the broader implications of the research results on the country's building stock energy usage.

First, the initial heating demand was extracted from this work, factoring in the uncertainty for each construction period. The specific area corresponding to each construction period was obtained from Streicher et al [22]. Subsequently, the heating demand per unit construction area, measured in kWh/a, was calculated.

Table S 7: Heating demand per construction period

Year of construction					
1911	1939	1960	1970	1972	1988
Construction period					
Before 1919	1919-1945	1946-1960	1961-1970	1971-1980	1981-1990
Heating demand (average), kWh/m^2,a					
175	104	135.7	187.6	106.8	125.8
Energy reference area per construction period (m^2)					
59200000	34700000	41000000	48900000	51300000	49700000
Heating demand per construction period (kWh/a)					
1.04E+10	3.61E+9	5.56E+09	9.17E+09	5.48E+09	6.25E+09

The heating type per construction period was extracted from the Federal Statistical office [23].

Table S 8: The number of buildings per each construction period

Period	before 1919	1919-1945	1946-1960	1961-1970	1971-1980	1981-1990
Oil	138497	97670	120892	128150	127517	89604
Gas	51237	41308	24362	13247	14163	30203
Electricity	30111	19659	17087	10662	30405	41387
Wood	98710	29331	16220	12136	10211	13672
Heat pump	10620	5650	5326	4340	8092	22327
Other heating type	9171	3851	4228	3986	4299	4103
Sum	338346	197469	188115	172521	194687	201296

Subsequently, the heating demand for each construction period and heating type was examined and analyzed.

Table S 9: Heating demand per heating type and construction period

Period	before 1919	1919-1945	1946-1960	1961-1970	1971-1980	1981-1990
Oil	4.24E+09	1.78E+09	3.58E+09	6.81E+09	3.59E+09	2.78E+09
Gas	1.57E+09	7.55E+08	7.21E+08	7.04E+08	3.99E+08	9.38E+08
Electricity	9.22E+08	3.59E+08	5.05E+08	5.67E+08	8.56E+08	1.29E+09
Wood	3.02E+09	5.36E+08	4.80E+08	6.45E+08	2.87E+08	4.25E+08
Heat pump	3.25E+08	1.03E+08	1.58E+08	2.31E+08	2.28E+08	6.93E+08
Other heating type	2.81E+08	7.04E+07	1.25E+08	2.12E+08	1.21E+08	1.27E+08

Taking into account the efficiency variations across different heating types (see Table S 10), the final energy consumption was computed (Table S 11).

Table S 10: Efficiency of the different heating systems

Heating type efficiency	
Oil boiler	0.91
Gas boiler	0.89
Electric boiler	0.5
Wooden boiler	0.8
Heat pump	4
Other heating type	0.9

Table S 11: Final energy demand considering the efficiency of the systems

End energy	before 1919	1919-1945	1946-1960	1961-1970	1971-1980	1981-1990
Oil	4.66E+09	1.96E+09	3.93E+09	7.49E+09	3.94E+09	3.06E+09
Gas	1.763E+09	0.85E+09	0.81E+09	0.79E+09	0.45E+09	1.05E+09
Electricity	1.844E+09	0.72E+09	1.01E+09	1.13E+09	1.7E+09	2.57E+09
Wood	3.778E+09	0.67E+09	0.6E+09	0.81E+09	0.36E+09	5.31E+08
Heat pump	0.81E+08	0.25E+08	0.39E+08	0.58E+08	0.57E+08	1.73E+08
Other heating type	3.1E+08	0.78E+08	1.4E+08	2.4E+08	1.3E+08	1.42E+08
Sum (kWh)	47.96E+09					
Sum (PJ)	172.7					

The total energy consumption, encompassing the construction periods and heating types, was juxtaposed against the energy demand of documented buildings by the Swiss Federal Office of Energy [24, p. 19]. The comparative analysis revealed 1.5% relative difference.

6. The resulting renovation options for conventional and non-conventional materials

The renovation solutions for the median values considering conventional and non-conventional materials can be seen in Table S 12 and Table S 13.

Table S 12: Optimal renovation solutions considering conventional materials

Building, year	Heating system	Windows	Exterior wall	Roof	Ground floor	Storebox (box above window with blinds)	LCCA, CHF/m^2,a	LCA kgCO$_2$eq/m^2,a	Qh, kWh/m^2,a
1, 1911	Gas	Current	Hagatherm 2cm Multipor 8cm	Cellulose fibre 21cm	Rockwool 11cm	-	6.57	22.38	94.5
	Wood pellets	Current	Hagatherm 2cm Multipor 8cm	Cellulose fibre 16.5cm	Rockwool 11cm	-	7.19	3.99	92.9
	Heat pump	Current	Hagatherm 2cm Multipor 8cm	Cellulose fibre 21cm	Rockwool 8cm	-	5.17	10.90	93.9
2, 1939	Gas	Current	Current	Glasswool 12 cm	Rockwool 8 cm	Glasswool 10 cm	6.64	19.44	50.6
	Wood pellets	Current	Current	Glasswool 12 cm	Rockwool 8 cm	Glasswool 11 cm	6.02	6.41	69.4
	Heat pump	Current	Current	Glasswool 12 cm	Rockwool 8 cm	Glasswool 12 cm	6.14	19.41	69.2
3, 1960	Gas	Wooden frame with double glazing	Wood fibre 10 cm	Current	Rockwool 8 cm	Glasswool 5 cm	13.16	33.45	50
	Wood pellets	Current	Current	Current	Rockwool 11 cm	Glasswool 4 cm	5.56	6.99	94.5
	Heat pump	Current	Current	Current	Rockwool 8 cm	Glasswool 7 cm	6.82	19.58	89.2
4, 1970	Gas	Wooden frame with double glazing	Wood fibre 10 cm	Glasswool 7 cm	Current	Glasswool 10 cm	9.40	21.36	66.2
	Wood pellets	Current	Wood fibre 10 cm	Glasswool 7 cm	Current	Glasswool 10 cm	10.74	7.71	64.9
	Heat pump	Current	10 cm wood fibre	Glasswool 7 cm	Current	Glasswool 10 cm	9.10	12.56	107.6
5, 1972	Gas	Current	Wood fibre 9.4 cm	Glasswool 23 cm	Rockwool 10 cm	Glasswool 7 cm	11.14	23.73	60.8
	Wood pellets	Current	Wood fibre 4.2 cm	Current	Rockwool 10 cm	Glasswool 7 cm	9.03	6.61	73.3
	Heat pump	Current	Current	Glasswool 16.1 cm	Rockwool 6.9 cm	Glasswool 12.7 cm	7.43	22.40	75.7

6, 1988	Gas	Wooden frame with double glazing	Wood fibre 7 cm	Current	Rockwool 10 cm	Glasswool 5 cm	8.35	19.10	59.2
	Wood pellets	Current	Wood fibre 7 cm	Current	Rockwool 7 cm	Glasswool 5 cm	6.08	6.30	76
	Heat pump	Current	Current	Rockwool 5 cm	Rockwool 7 cm	Glasswool 8 cm	4.64	12.28	76.2

Table S 13: Optimal renovation solutions considering non-conventional materials

Building, year	Heating system	Windows	Exterior wall	Roof	Ground floor	Storebox (box above window with blinds)	LCCA, CHF/m^2,a	LCA kgCO$_2$eq/m^2,a	Qh, kWh/m^2,a
1, 1911	Gas	Current	Straw bale 70 cm	Straw bale 70 cm	EPS 20 cm	-	6.54	13.84	44.1
	Wood pellets	Current	Straw bale 70 cm	Straw bale 70 cm	EPS 10 cm	-	5.08	1.89	48.6
	Heat pump	Current	Straw bale 70 cm	Straw bale 70 cm	Hempcrete 38 cm	-	4.72	2.72	47.2
2, 1939	Gas	Wooden frame with double glazing	Straw bale 70 cm	Straw bale 70 cm	Hempcrete 38 cm	Straw bale 70 cm	4.61	7.32	35.9
	Wood pellets	Current	Straw bale 70 cm	Straw bale 70 cm	Hempcrete 38 cm	Straw bale 70 cm	5.91	1.08	32.3
	Heat pump	Current	Straw bale 70 cm	Straw bale 70 cm	Hempcrete 38 cm	Straw bale 70 cm	4.69	1.61	35.2
3, 1960	Gas	Current	Straw bale 70 cm	Straw bale 70 cm	Hempcrete 38 cm	Hemp mat 22 cm	7.73	20.55	40.3
	Wood pellets	Current	Straw bale 70 cm	Straw bale 70 cm	Hempcrete 38 cm	Straw bale 70 cm	4.26	1.21	40.1
	Heat pump	Current	Straw bale 70 cm	Straw bale 70 cm	Hempcrete 38 cm	Hempcrete 8 cm	5.37	1.66	36.8
4, 1970	Gas	Current	Straw bale 70 cm	Straw bale 70 cm	EPS 20 cm	Wood fibre 6 cm	4.49	7.97	45.2
	Wood pellets	Current	Hemp mat 12 cm	Straw bale 70 cm	Hempcrete 38 cm	Straw bale 70 cm	5.53	2.41	45.4
	Heat pump	Current	Straw bale 70 cm	Straw bale 70 cm	EPS 15 cm	Wood fibre 10 cm	3.85	2.95	49.1
5, 1972	Gas	Current	Hemp mat 18 cm	Hemp mat 18 cm	EPS 10 cm	Straw bale 70 cm	6.31	14.73	58.7
	Wood pellets	Current	Straw bale 20 cm	Straw bale 70 cm	EPS 15 cm	Straw bale 70 cm	7.16	2.99	39.4
	Heat pump	Current	Straw bale 70 cm	Hemp mat 22 cm	EPS 10 cm	Straw bale 70 cm	8.64	3.24	38.7
6, 1988	Gas	Current	Straw bale 70 cm	Straw bale 48 cm	EPS 20 cm	Straw bale 20 cm	9.59	15.77	44.8
	Wood pellets	Current	Straw bale 70 cm	Straw bale 70 cm	Hempcrete 38 cm	Wood fibre 12 cm	4.96	1.34	44.6
	Heat pump	Current	Straw bale 48 cm	Straw bale 70 cm	Hempcrete 20 cm	Hemp mat 6 cm	3.96	1.85	47.8

7. Initial energy consumption and LCA, LCCA and insulation thickness of the renovated solutions for gas and heat pump heating systems

In the following figures, the results for the LCA, LCCA and insulation thickness for the gas boiler and heat pump are shown.

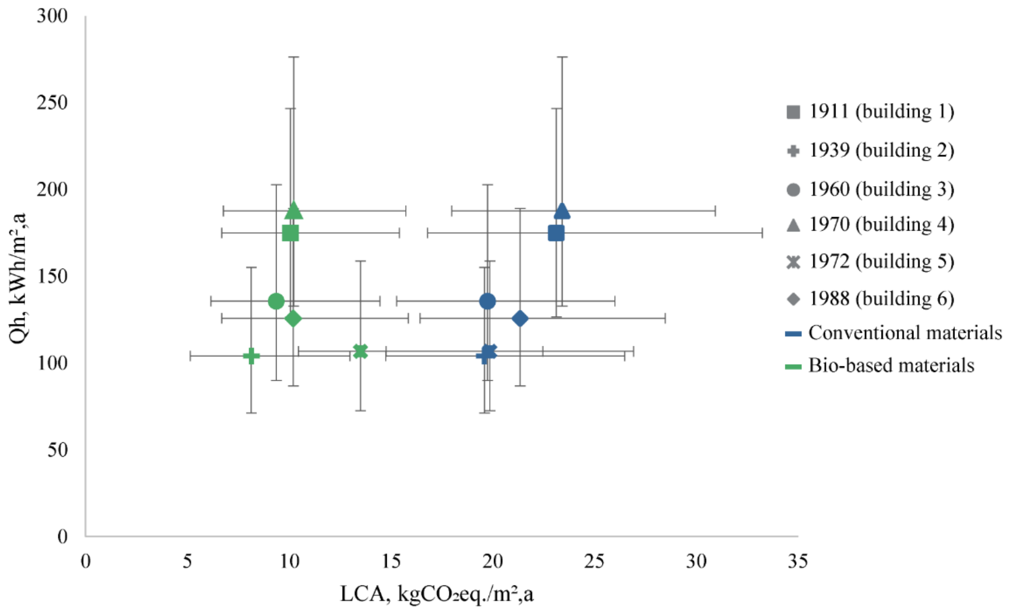

Figure S 1: Initial energy consumption and renovated results of LCA considering non-conventional and conventional materials for gas boiler

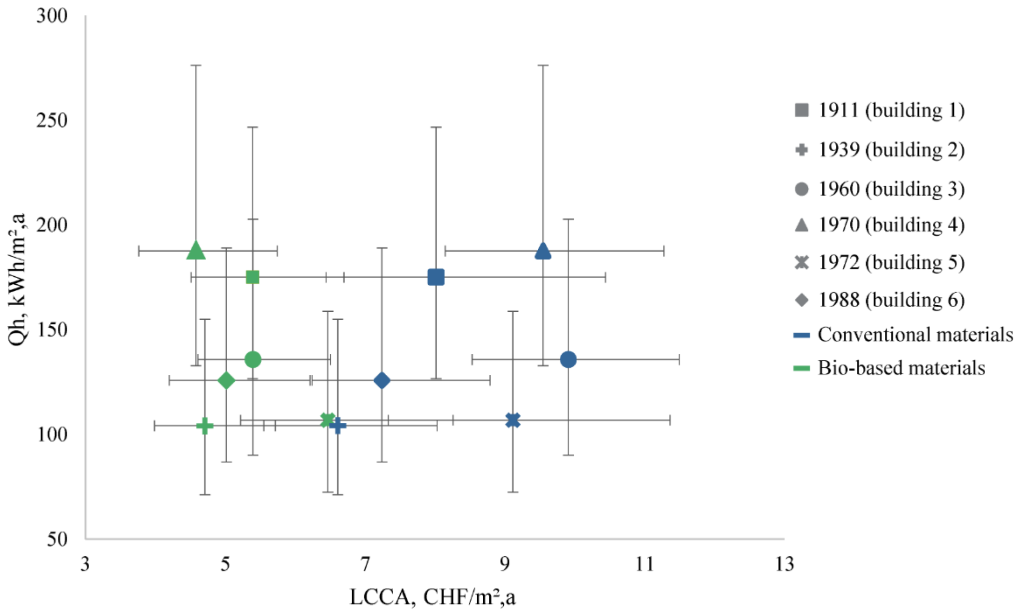

Figure S 2: Initial energy consumption and renovated results of LCCA considering non-conventional and conventional materials for gas boiler

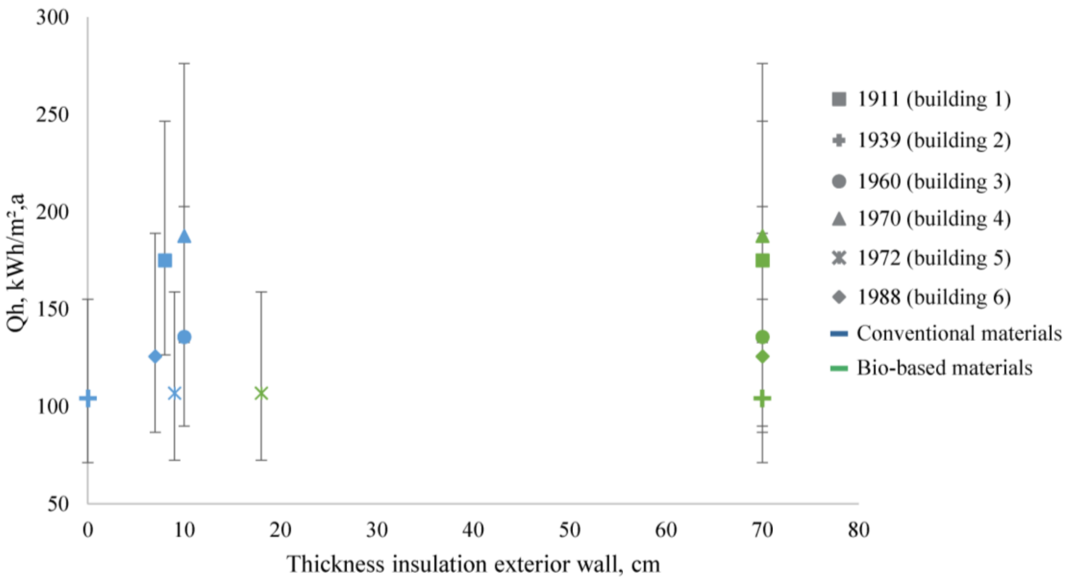

Figure S 3: Initial energy consumption and thickness of thermal insulation after renovation considering non-conventionaland conventional materials for gas boiler

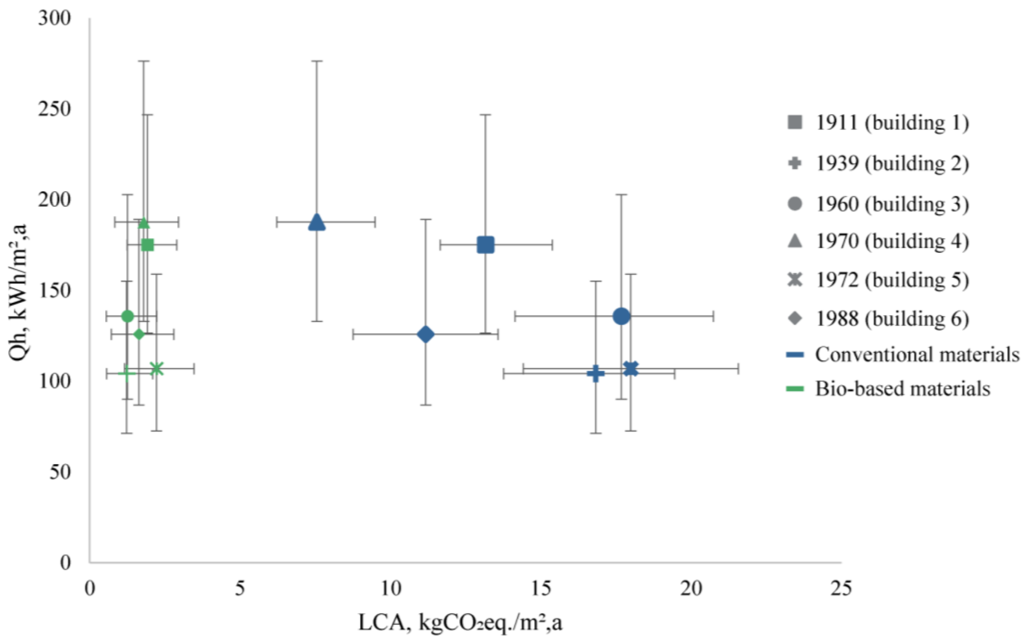

Figure S 4: Initial energy consumption and renovated results of LCA considering non-conventionaland conventional materials for heat pump

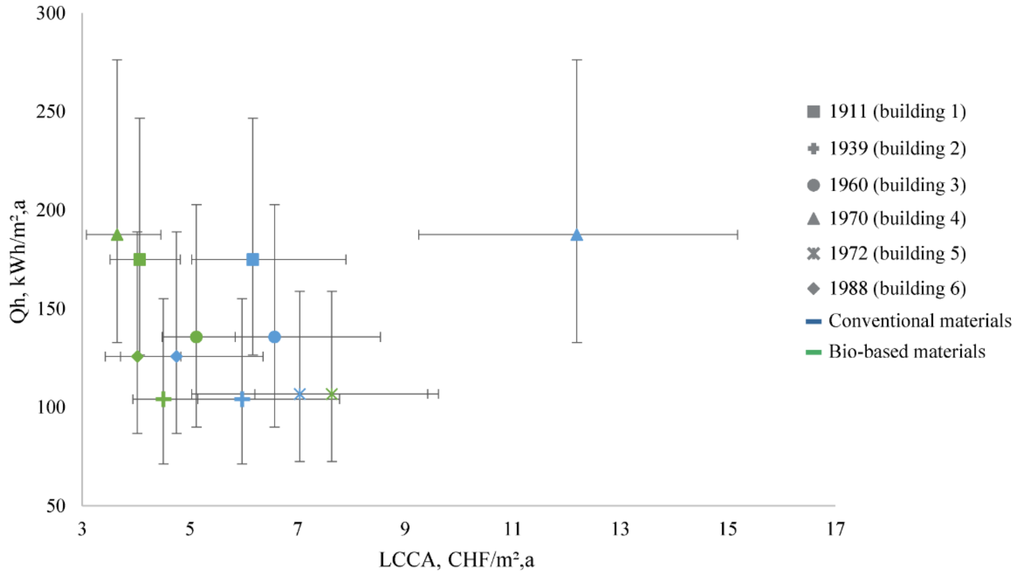

Figure S 5: Initial energy consumption and renovated results of LCCA considering non-conventionaland conventional materials for heat pump

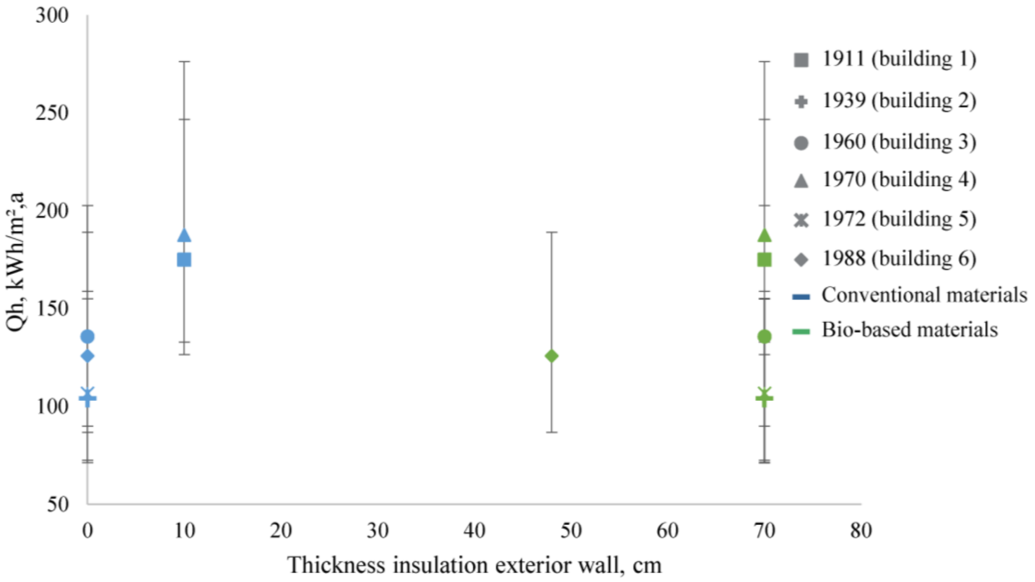

Figure S 6: Initial energy consumption and thickness of thermal insulation after renovation considering non-conventionaland conventional materials for heat pump

8. Electricity mix scenarios

To account for uncertainties in the electricity mix, three scenarios have been introduced (Figures S 1 and S 2). The configurations depicted in the figures represent the post-nuclear phase-out scenarios. In Variant C, the forthcoming electricity supply deficit is supplied by gas. In Variant C+E, the deficit is met through a combination of combined cycle gas power plants and renewable energy sources. Variant E fills the future energy supply gap with renewable energy sources and imports. The comprehensive procedure for conducting scenario analysis is outlined in Schurtenberger and Zhou [25]. Notably, the scenarios distinguish between summer and winter seasons due to variations in import ratios.

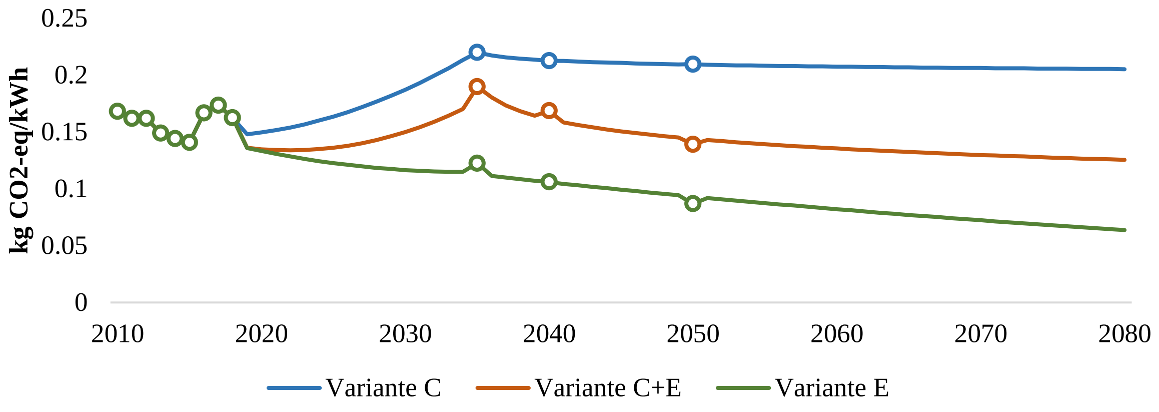

Figure S 7: Electricity mix scenarios for environmental impacts attributed to winter season

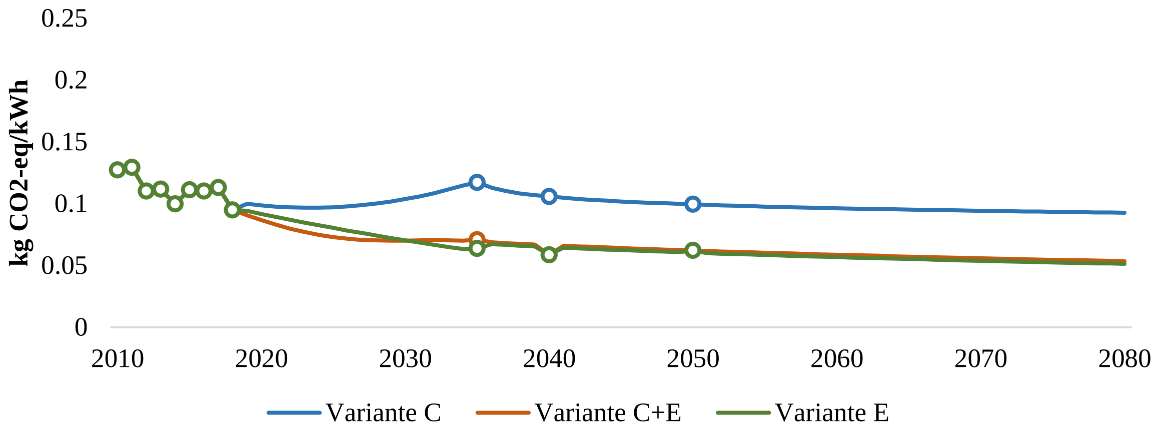

Figure S 8: Electricity mix scenarios for environmental impacts attributed to summer season

The associated electricity costs are shown in Figure S 9, utilizing data sourced from prospective estimations of varying energy type costs in Switzerland.

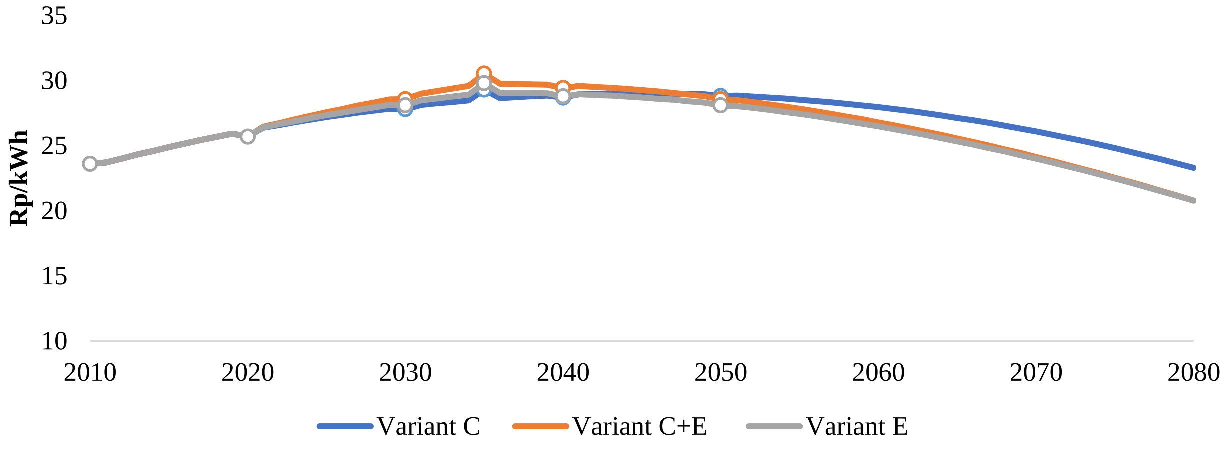

Figure S 9: Electricity mix scenarios for costs

References:

[1] G. Wernet, C. Bauer, B. Steubing, J. Reinhard, and B. Moreno-Ruiz, E., Weidema, “The

ecoinvent database version 3 (part I): overview and methodology," *Int. J. Life Cycle Assess.*, vol. 21(9), p. pp.1218–1230, 2016.

[2] WWF, "Vergleich der Jahreskosten und Umweltauswirkungen von Heizungssystemen." 2015.

[3] SIA 480, "Wirtschaftlichkeitsrechnung für Investitionen im Hochbau," 2016.

[4] A. Säynäjoki, J. Heinonen, S. Junnila, and A. Horvath, "Can life-cycle assessment produce reliable policy guidelines in the building sector?," *Environ. Res. Lett.*, vol. 12, no. 1, 2017.

[5] C. Chen, G. Habert, Y. Bouzidi, and A. Jullien, "Environmental impact of cement production: detail of the different processes and cement plant variability evaluation," *J. Clean. Prod.*, vol. 18, no. 5, pp. 478–485, 2010.

[6] M. Toczé, "Interior renovation solutions for historical buildings," University of applied sciences Upper Austria; INSA Strassbourg, 2018.

[7] State Secretariat for economic Affairs (SECO), "Economic forecasts," 2019. [Online]. Available: https://www.seco.admin.ch/seco/en/home/wirtschaftslage---wirtschaftspolitik/Wirtschaftslage/konjunkturprognosen.html.

[8] S. Lasvaux *et al.*, "DUREE Project, Analysis of lifetimes of building elements in the literature and in renovation practices and sensitivity analyses on building LCA & LCC," no. October, 2019.

[9] D. Fernandes, J. de Brito, and A. Silva, "Methodology for service life prediction of window frames," *Can. J. Civ. Eng.*, vol. 46, no. 11, pp. 1010–1020, 2019.

[10] F. Domínguez-Muñoz, B. Anderson, J. M. Cejudo-López, and A. Carrillo-Andrés, "Uncertainty in the thermal condusctivity of insulation materials," in *Eleventh International IBPSA Conference*, 2009, pp. 1008–1013.

[11] European comission directorate-general for energy, "Mapping and analyses of the current and future (2020 - 2030) heating/cooling fuel deployment (fossil/renewables)," 2016.

[12] V. R. Gharehbaghi, A. Nguyen, E. Noroozinejad Farsangi, and T. Y. Yang, "Supervised damage and deterioration detection in building structures using an enhanced autoregressive time-series approach," *J. Build. Eng.*, vol. 30, no. December 2019, p. 101292, 2020.

[13] J. Khoury, P. Hollmuller, B. Lachal, and U. Schneider, Stefan Lehmann, "COMPARE RENOVE : du catalogue de solutions à la performance réelle des rénovations énergétiques," 2018.

[14] SIA 380/1, "Heizwärmebedarf." 2016.

[15] H. G. Beyer and B. Sendhoff, "Robust optimization - A comprehensive survey," *Comput. Methods Appl. Mech. Eng.*, vol. 196, no. 33–34, pp. 3190–3218, 2007.

[16] C. Zang, M. I. Friswell, and J. E. Mottershead, "A review of robust optimal design and its application in dynamics," *Comput. Struct.*, vol. 83, no. 4–5, pp. 315–326, 2005.

[17] M. Moustapha, B. Sudret, J. M. Bourinet, and B. Guillaume, "Quantile-based optimization under uncertainties using adaptive Kriging surrogate models," *Struct. Multidiscip. Optim.*, vol. 54, no. 6, pp. 1403–1421, 2016.

[18] K. Deb, A. Pratap, S. Agarwal, and T. Meyarivan, "A fast and elitist multiobjective genetic algorithm: NSGA-II," *IEEE Trans. Evol. Comput.*, vol. 6, no. 2, pp. 182–197, 2002.

[19] C. E. Rasmussen and C. K. I. Williams, *Gaussian processes for machine learning (Internet ed.). Adaptive computation and machine learning*. Cambridge, Massachusetts: MIT Press., 2006.

[20] T. J. Santner, B. J. Williams, and W. I. Notz, *The Design and Analysis of Computer*

Experiments, Second. Springer, 2018.

[21] M. Moustapha, A. Galimshina, G. Habert, and B. Sudret, "Surrogate-assisted multi-objective robust optimization with application to problems with mixed continuous-categorical parameters," pp. 1–20.

[22] K. N. Streicher, P. Padey, D. Parra, M. C. Bürer, S. Schneider, and M. K. Patel, "Analysis of space heating demand in the Swiss residential building stock: Element-based bottom-up model of archetype buildings," *Energy Build.*, vol. 184, pp. 300–322, 2019.

[23] Federal Statistical office, "The main energy sources for heating by period of construction of buildings," 2015. [Online]. Available: https://www.bfs.admin.ch/bfs/en/home/statistics/catalogues-databases/graphs.assetdetail.3182092.html.

[24] A. Kemmler, T. Trachsel, and P. Vu, "Der Energieverbrauch der Privaten Haushalte Energieverbrauch der Privaten Haushalte 2000 – 2021," 2022.

[25] S. Schurtenberger and S. Zhou, "Prospective technology evolution in dynamic life cycle assessment," Zurich, 2019.
